# Resolving *Salmonella* infection reveals dynamic and persisting changes in murine bone marrow progenitor cell phenotype and function

**DOI:** 10.1002/eji.201344350

**Published:** 2014-06-11

**Authors:** Ewan A Ross, Adriana Flores-Langarica, Saeeda Bobat, Ruth E Coughlan, Jennifer L Marshall, Jessica R Hitchcock, Charlotte N Cook, Manuela M Carvalho-Gaspar, Andrea M Mitchell, Mary Clarke, Paloma Garcia, Mark Cobbold, Tim J Mitchell, Ian R Henderson, Nick D Jones, Graham Anderson, Christopher D Buckley, Adam F Cunningham

**Affiliations:** MRC Centre for Immune Regulation, Institute for Microbiology and Infection, School of Immunity and Infection, Institute for Biomedical Research, Medical School, University of BirminghamEdgbaston, Birmingham, UK

**Keywords:** Bacterial Infection, Bone Marrow, Leucopoiesis, Progenitor, *Salmonella*

## Abstract

The generation of immune cells from BM precursors is a carefully regulated process. This is essential to limit the potential for oncogenesis and autoimmunity yet protect against infection. How infection modulates this is unclear. *Salmonella* can colonize systemic sites including the BM and spleen. This resolving infection has multiple IFN-γ-mediated acute and chronic effects on BM progenitors, and during the first week of infection IFN-γ is produced by myeloid, NK, NKT, CD4^+^ T cells, and some lineage-negative cells. After infection, the phenotype of BM progenitors rapidly but reversibly alters, with a peak ∼30-fold increase in Sca-1^hi^ progenitors and a corresponding loss of Sca-1^lo/int^ subsets. Most strikingly, the capacity of donor Sca-1^hi^ cells to reconstitute an irradiated host is reduced; the longer donor mice are exposed to infection, and Sca-1^hi^c-kit^int^ cells have an increased potential to generate B1a-like cells. Thus, *Salmonella* can have a prolonged influence on BM progenitor functionality not directly related to bacterial persistence. These results reflect changes observed in leucopoiesis during aging and suggest that BM functionality can be modulated by life-long, periodic exposure to infection. Better understanding of this process could offer novel therapeutic opportunities to modulate BM functionality and promote healthy aging.

## Introduction

Under steady-state conditions, new innate and adaptive immune cells are generated by hematopoiesis in the bone marrow (BM). They develop from hematopoietic stem cells (HSCs) that differentiate into progenitor cells that acquire mature lineage (Lin) markers. These progenitor populations can be identified by their lack of expression of mature Lin markers (such as CD5, Ter119, B220, CD11b, Gr-1) and their varying expression of Sca-1 and c-Kit (LSK) 1. In the steady state, the least differentiated quiescent long-term reconstituting HSCs (LT-HSCs) sit in stromal BM niches, before being activated to proliferate and differentiate to short-term reconstituting HSCs (ST-HSCs) and multipotent progenitors (MPPs) to provide the precursors of blood and immune cells 2,3. Infection can have a profound impact on the BM, in part reflecting the capacity of this site to be directly colonized by the organism. An important concept that arises from studying the impact of infections on the BM is that the cellular constitution of the BM is not static but can alter, resulting in the activation and expansion of the HSC compartment 4–8. Nevertheless, despite some understanding of the effects of infection on the BM progenitor populations, it remains unclear how long these effects on BM progenitors last for during infection and whether they remain after the pathogen has been cleared.

*Salmonella enterica* serovar Typhimurium (STm) can cause devastating systemic infections. Primary, systemic infection of Nramp's strains of mice, such as C57BL/6 and BALB/c, with virulent strains of STm is overwhelming and lethal within a matter of days and is not representative of natural infection. Use of attenuated strains of STm can result in a disseminated, yet ultimately, resolving infection that fits many of the observed features of systemic salmonellosis. Clearance of bacteria requires CD4^+^ T cells and the generation and persistence of a Th1-polarized response 9–11. During primary infection, STm colonizes sites such as the spleen, liver, and BM, inducing a splenomegaly that is due to both an accumulation of leukocytes and erythropoiesis switching to the spleen from the BM 12–14. Splenomegaly reduces as infection is cleared, but never resolves to its size as in preinfection, resulting in a chronic alteration of the erythropoietic, myeloid, and lymphoid cellular constitution of the spleen, even years postinfection 12. Thus, STm infection can result in short- and long-term effects on cell populations in organs such as the spleen that persist long after bacteria have been cleared. These findings reflect our studies on T-cell survival during infection. These studies showed that naïve CD4^+^ T-cell numbers were maintained even during increased infection-associated activation-induced cell death and thymic atrophy 11,15,16. Thymic output of mature T cells was maintained despite a >20-fold reduction in the total cellularity of the thymus. Since there is an obvious link between thymic function and the BM, we examined the impact of STm infection on the BM, with a particular focus on Lin^−^ hematopoietic progenitor populations. This showed how there were reversible changes in the phenotypic constitution of the Lin^−^ population in the BM that depended upon the stage of infection. Most significantly, the ability of progenitor cells that were isolated from mice infected for different lengths of time exhibited an altered reconstitution potential, which had a particular impact on lymphocyte reconstitution. This work has implications for understanding immune homeostasis during infection and for how infection may influence the skew to myeloid cell production seen during aging 17–20.

## Results

### Systemic STm infection alters BM cellularity

**Figure 1:**
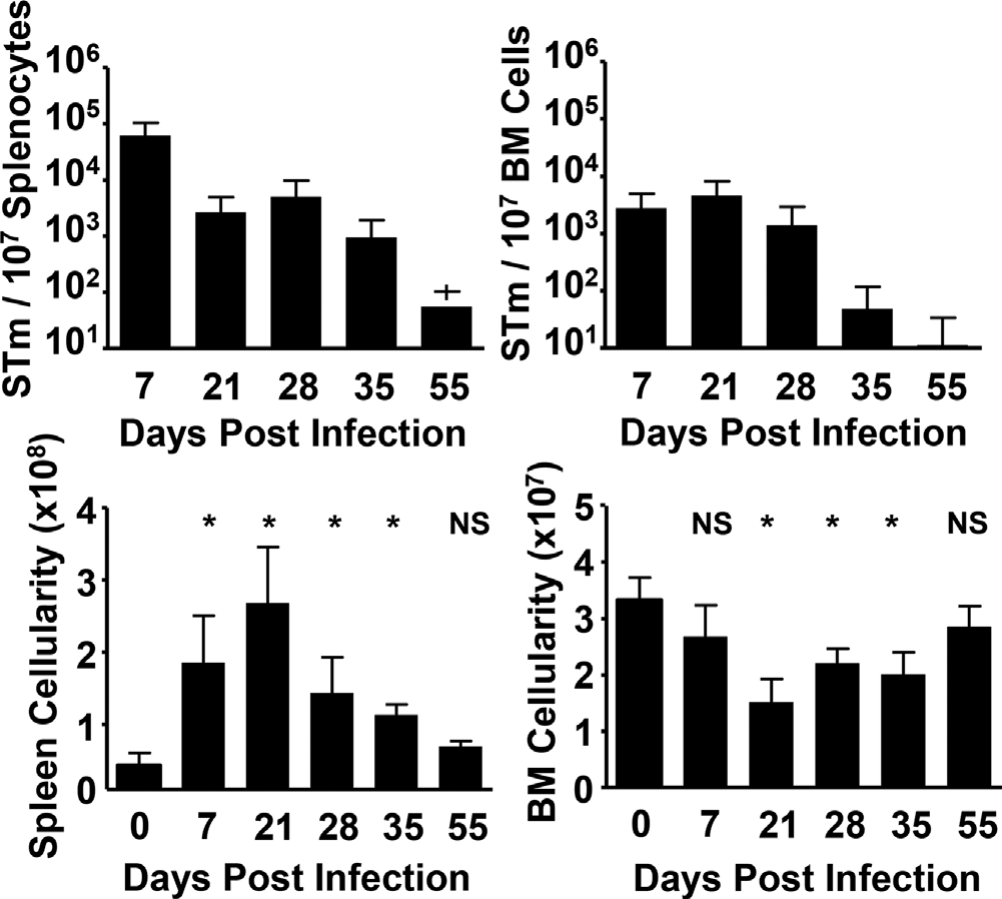
Infection with STm results in rapid BM colonization and a reduction in cellularity that recovers upon clearance of systemic bacterial burden. WT mice were immunized i.p. with 5 × 10^5^ STm, and bacterial burden in the spleen and BM of mice was quantified at key time-points during infection. The impact of colonization on splenic and BM homeostasis was quantified by measurement of changes to total cellularity in these tissues. Data are shown as mean + S.D. (*n* = 4) and are representative of at least three independent time-courses. *p ≤ 0.05 or nonsignificant (NS) compared to noninfected day 0 controls; two-tailed student's *t*-test.

To assess the impact of infection on the BM, C57BL/6 WT mice were infected i.p. with 5 × 10^5^ STm. In this systemic infection, bacteria colonize sites such as the spleen and BM. Clearance of STm from the BM occurred with similar kinetics to the spleen, and bacteria were undetectable in the BM by day 70 postinfection (Fig.1 and data not shown). Although infection results in a substantial increase in splenocyte numbers, the impact on BM cellularity is less marked. Numbers of BM cells fell after infection, being approximately twofold lower at their nadir on day 21, before recovering as infection was cleared (Fig.1). Therefore STm infection has distinct impacts on the cellularity of differing sites of colonization.

### Phenotypic analysis of Lin^−^ cells reveals dramatic, reversible, effects on BM cell homeostasis

**Figure 2:**
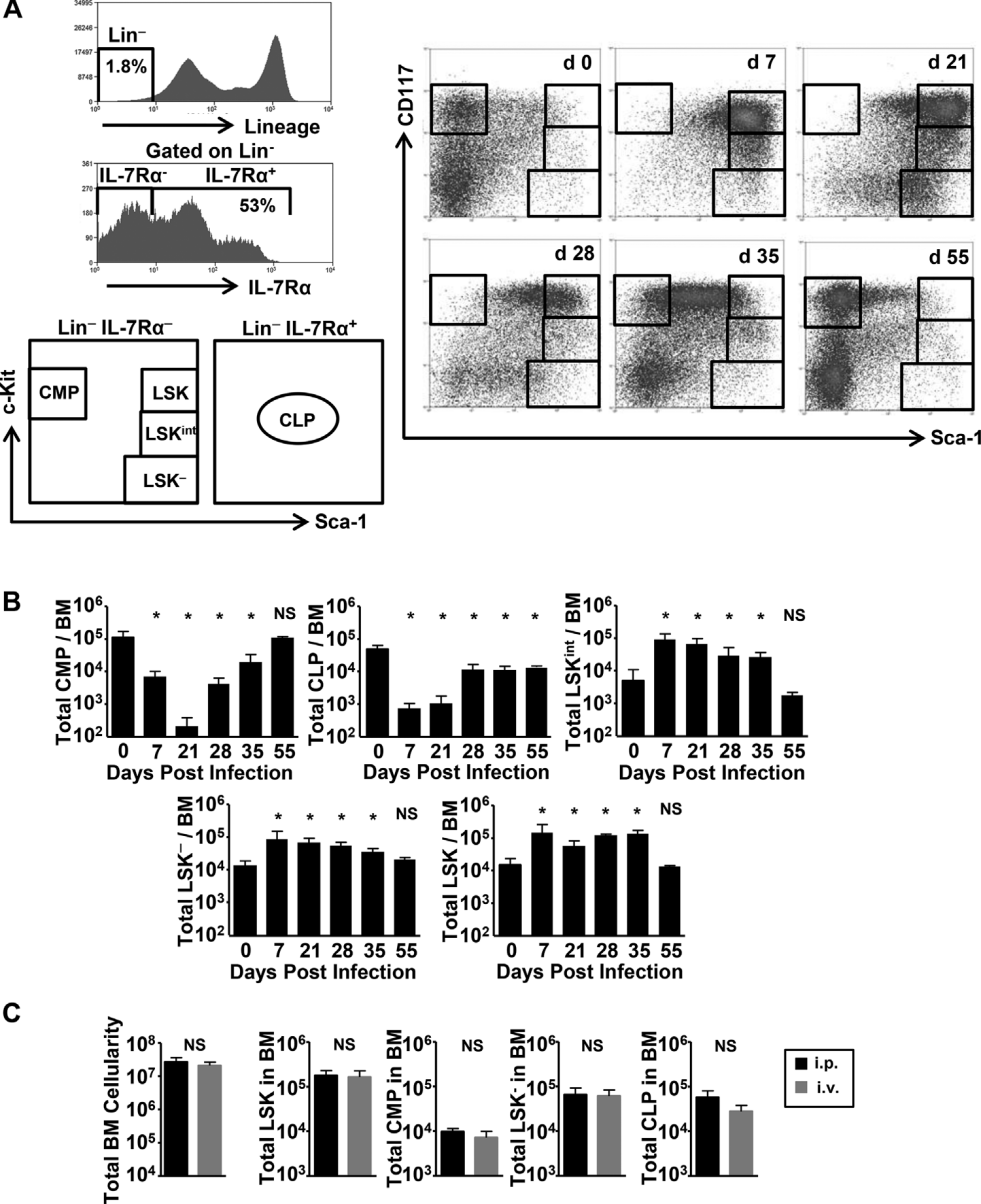
STm infection and colonization of the BM results in rapid, reversible changes to progenitor populations. (A) BM from WT mice were collected at 0, 7, 21, 28, 35, and 55 days postSTm immunization and progenitor populations identified by flow cytometry as shown in the representative histograms and plots, using combinations of IL-7Rα, Sca-1, and c-Kit, and lack of expression of mature lineage (Lin) markers. (B) Graphs show the absolute number of identified progenitor populations in the total BM by flow cytometry. (C) C57BL/6 mice were challenged with 5 × 10^5^ STm either i.p. or i.v. for 7 days and BM progenitor subsets quantified as in (A). Data are shown as mean + S.D. (*n* = 4) and are representative of at least three independent time-courses in (B) and two independent experiments in (C). *p ≤ 0.05 or nonsignificant (NS) compared to noninfected day 0 controls in (B) or between groups in (C); two-tailed student's *t*-test.

A major function of the BM is to generate leukocytes from Lin^−^ precursors. The progenitor populations of these typically constitute <1% of total BM cell numbers. Since infection had only modest effects on total BM cell numbers, we examined its effects on progenitor populations by flow cytometry using standard markers (Fig.2A, 21,22). This showed that infection resulted in dramatic changes in Lin^−^ cells based on c-Kit and Sca-1 expression (Fig.2B). These changes are explained in detail below but were most associated with a loss of Sca-1^lo/int^ populations and an increase in Sca-1^hi^ populations, with or without associated changes in c-Kit expression. These effects were not dependent upon the route of infection as mice infected i.p. or i.v. showed similar alterations (Fig.2C).

Changes in BM Lin^−^ populations were rapid and detectable by 24 h postinfection (data not shown). The changes persisted throughout infection and were reversible, since the Lin^−^ BM phenotype returned to a resting state resembling that of noninfected mice by day 55 (Fig.2A). In particular, at day 21 postinfection there was a >90% loss in common myeloid progenitors (CMP; as defined by Lin^−^c-Kit^+^Sca-1^−^ and IL-7Rα^−^) and common lymphoid progenitors (CLPs; as defined by Lin^−^IL-7Rα^+^c-Kit^int^Sca-1^int^) (Fig.2B). Finally, these changes were not obviously directly related to the total bacterial burden in the BM as bacterial numbers were generally static when these changes were ongoing (Fig.1 and2B).

### IFN-γ, but not TLR4, is required for infection induced changes to BM progenitors

**Figure 3:**
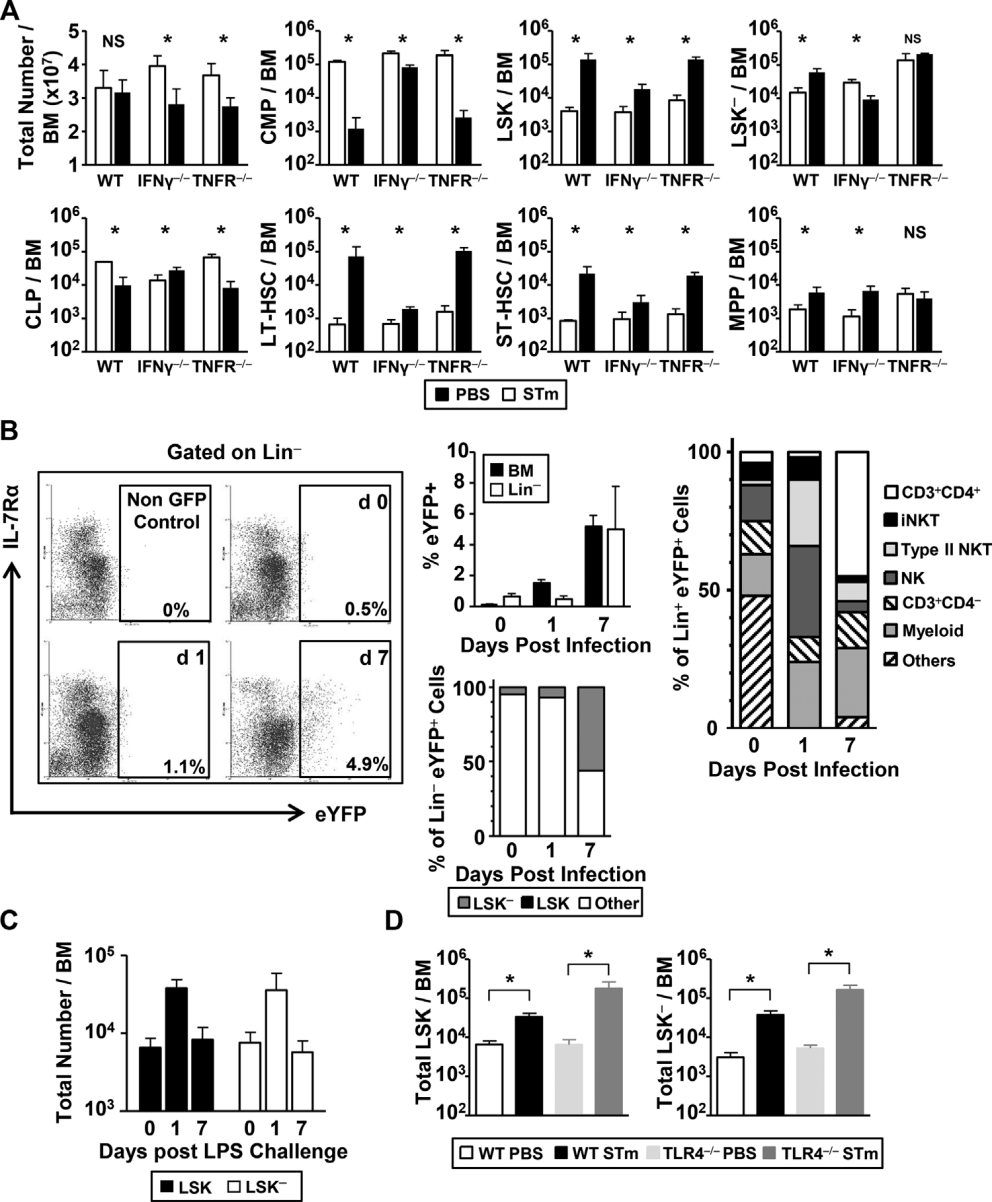
The early BM response to *Salmonella* requires IFN-γ but is independent of TLR4. (A) WT, IFN-γ or TNF-R1, and R2 (TNFR^−/−^) deficient mice were challenged with PBS or STm for 7 days, and changes to BM progenitor population numbers were assessed by flow cytometry. (B) IFN-γ-EYFP reporter mice were challenged with 5 × 10^5^ STm for 1 or 7 days and EYFP expression in the Lin^−^ fraction analyzed by flow cytometry as shown in the representative plots. Top left graph shows the proportion of IFN-γ-EYFP expressing cells in the whole BM or Lin^−^ fraction. Cells producing IFN-γ were identified by flow cytometry in either the whole BM (right graph) or the Lin^−^ fraction (bottom left graph). (C) WT mice were challenged with 20 μg purified LPS i.p. and changes to BM progenitor numbers identified by flow cytometry at day 1 and 7 post-challenge. (D) WT or TLR4^−/−^ mice were challenged with either STm or PBS for 7 days and changes to progenitor populations quantified by flow cytometry. (A–D) Data are shown as mean + S.D. (*n* = 4) and are representative of two independent experiments.*p ≤ 0.05 or nonsignificant (NS); two-tailed student's *t*-test.

STm infection induces the release of cytokines such as IFN-γ and TNF-α. Sca-1 expression on BM progenitors can increase after exposure to IFN-γ, although their expansion can be modulated by TNF-α 4,5,7,8,23–25. To assess whether these cytokines were involved in the early changes in the BM after STm infection, we infected IFN-γ or TNF-αR deficient mice and assessed changes to BM progenitors by flow cytometry (Fig.3A). There was a greater requirement for IFN-γ, than for TNF-α signaling, for modulating BM progenitor numbers. To identify which BM cells produced IFN-γ during the early response to STm, IFN-γ-eYFP reporter mice were infected and eYFP positive cells were identified by flow cytometry (Fig.3B). In the absence of infection, a few cells in the BM expressed eYFP and those that did expressed it weakly (representative flow cytometry shown for Lin^−^ cells in Fig.3B). By day 1 after infection, there was a >10-fold increase in the proportion of eYFP^+^ BM cells, and this signal was largely detected in cells that had an iNK T-cell-like phenotype (TCRβ^+^PBS-57 tetramer^+^) or that were NK1.1^+^ and tetramer^−^. Of the NK1.1^+^ tetramer^−^ population, approximately 35% were type II NKT cells (CD3^+^NK1.1^+^PBS-57 tetramer^−^), the remainder were NK cells. On day 7, the predominant cells producing eYFP were classical CD4^+^ T-cells that did not express NK1.1 or bind tetramer and CD11b or CD11c myeloid cells (Fig.3B).

Less than 1% of Lin^−^ BM cells were eYFP^+^ at baseline or day 1, although this rose markedly by day 7. The majority of the eYFP expression was detected in the Lin^−^ cells within the nonLSK or LSK^−^ populations at day 0 and day 1, although by day 7 there was a marked expansion of eYFP^+^ LSK^−^ cells (Fig.3B).

An obvious candidate bacterial antigen that could drive these changes in the Lin^−^ populations is LPS and immunization of mice with 20 μg of LPS resulted in an increase in LSK and LSK^−^ cells at day 1, although numbers returned to normal by day 7 (Fig.3C). Nevertheless, the response observed after STm is likely to be more complicated than a simple response to LPS as infection of mice lacking TLR4 resulted in a similar increase in LSK and LSK^−^ cells to that observed in WT mice (Fig.3D).

### *Salmonella* Infection results in the expansion of atypical BM progenitor populations

**Figure 4:**
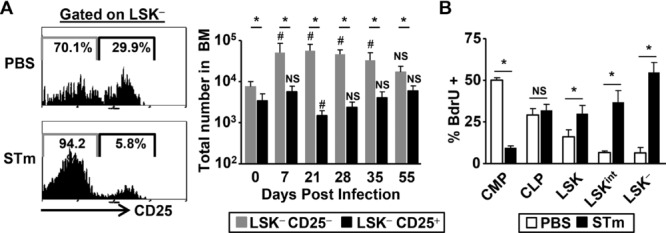
*Salmonella* induced increase in BM LSK^−^ population occurs through expansion of distinct subsets and induced proliferation. (A) Representative histograms show the expression of CD25 on LSK^−^ cells from PBS or STm infected BM of WT mice. Graph shows the total numbers of CD25^+^ and CD25^−^ LSK^−^ cells in the BM during a representative time-course of STm infection. (B) After 7 days postadministration of STm or PBS, actively proliferating BM progenitors were quantified by BrdU uptake and flow cytometry. (A and B) Data are shown as mean + S.D. (*n* = 4) and are representative of two independent (A) time-courses or (B) experiments. *p ≤ 0.05 or nonsignificant (NS) of LSK^−^CD25^+^ compared to LSK^−^CD25^−^; ^#^*p* ≤ 0.05 or NS of CD25 subset numbers compared to day 0; *p ≤ 0.05 or NS compared to PBS controls; two-tailed student's *t*-test.

Infection induced a dramatic increase in three BM Lin^−^ subsets that are only minority populations in noninfected mice (Fig.2A). First, there was a tenfold increase in the stem cell containing Lin^−^ Sca-1^+^c-Kit^+^ (LSK) population by 7 days postinfection, which remained elevated compared to noninfected mice until after 35 days postinfection. Second, there was a similar fold increase in the Lin^−^ Sca-1^+^c-Kit^−^ (LSK^−^) population, which also peaked by 7-day postinfection before falling gradually to preinfection levels by day 55. In the steady state this small, reportedly quiescent and heterogeneous, population has been described to contain both lymphoid and myeloid progenitors 26–28 and can be subdivided based on CD25 expression (Fig.4A, 29). Although both subsets are present in noninfected mice, the CD25^−^ subset expands dramatically after infection, whereas the LSK^−^CD25^+^ numbers remain relatively constant. Further analysis revealed that increased number of the LSK^−^ population primarily reflected the expansion of the IL-7Rα and Flt3 double-negative CD25^−^ population, with other populations showing more variable changes (Supporting Information Fig. 1).

Finally, and most dramatically, by day 7 there was a 30-fold increase in a population of cells expressing intermediate levels of c-Kit (LSK^int^; Lin^−^Sca-1^+^c-Kit^int^). From day 7 to when infection resolved the three Sca-1^hi^ populations constituted up to 80% of the total Lin^−^ fraction compared to 10% before infection (Supporting Information Fig. 2). A BrdU pulse 2 h before sacrifice showed that after infection all Sca-1^hi^ fractions contained a higher proportion of proliferating cells, with levels of proliferation highest in the LSK^−^ cells (Fig.4B). In contrast, proliferation was not enhanced in conventionally defined CMP or CLP populations, and the increase in Sca-1^hi^ populations was not associated with the upregulation of Sca-1 expression on CMP as defined using conventional markers (30, Fig.2B). Once bacterial clearance was nearly complete, the BM Lin^−^ populations returned to a phenotype that resembled noninfected controls. Thus, STm infection drives substantial, reversible alterations in the phenotype of BM Lin^−^ progenitor populations.

### Bacterial infection induces HSC activation and expansion within the c-Kit^hi^Sca-1^hi^ LSK population

**Figure 5:**
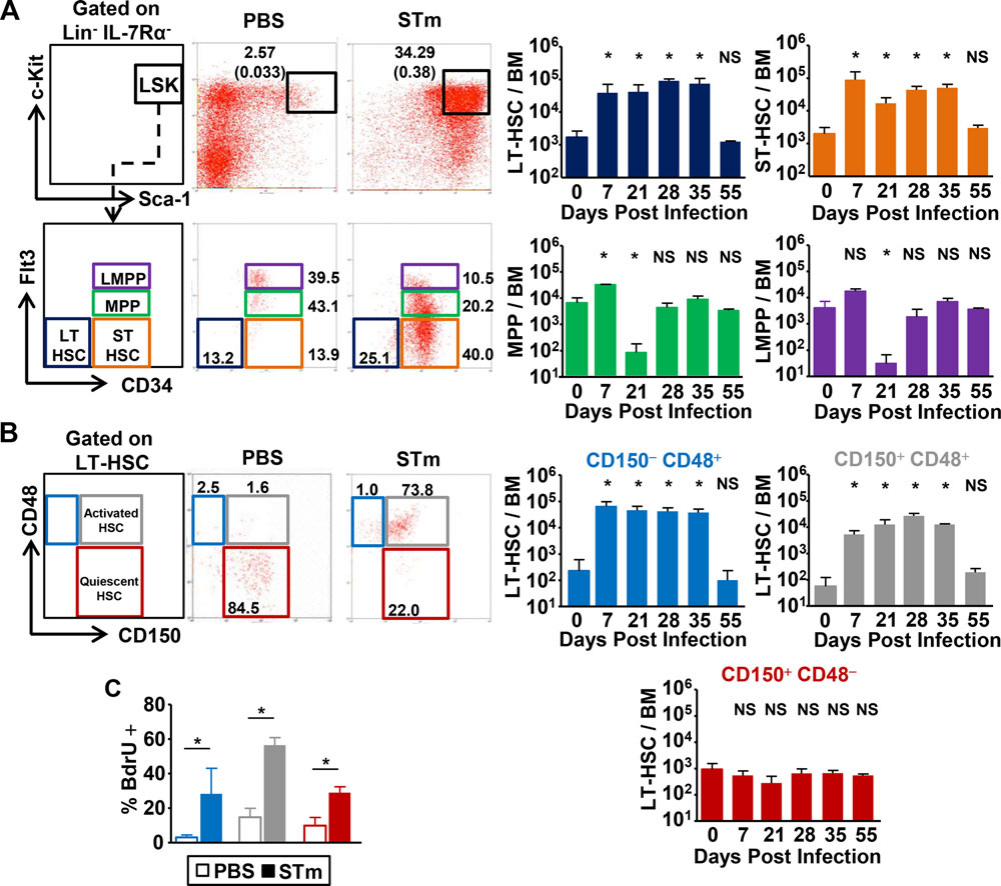
Infection with STm drives the expansion of the LSK population and activation of LT-HSCs. (A) WT mice were infected with STm, and the BM LSK population was further subdivided by flow cytometry into LT-HSCs, ST-HSCs, and MPPs using the expression of CD34 and Flt3. Numbers in the top panels depict the LSK population as a proportion of the lineage negative fraction, and in brackets of the whole BM. In the lower panels, numbers depict the individual subsets as a proportion of the total LSK population. Graphs on right show the absolute numbers of these fractions in the BM during the time-course of infection. (B) Representative flow cytometry plots of CD150 and CD48 expression on LT-HSCs from WT mice immunized with PBS or STm for 7 days. Numbers depict the proportion of the subsets as a percentage of total LT-HSC cells. Graphs show the total number of each fraction identified in the BM. (C) After 7 days exposure to STm, the proportion of LT-HSC populations that were actively proliferating was quantified by BrdU uptake and flow cytometry. (A–C) Data are shown as mean + S.D. (*n* = 4) and (A and B) are representative of at least three independent time-courses or (C) two independent experiments. *p ≤ 0.05 or nonsignificant (NS) compared to day 0 noninfected or PBS controls; two-tailed student's *t*-test.

As infection resulted in increase in the three Sca-1^hi^ populations, we examined these in greater depth. Based on previous studies examining CD34 and Flt3 expression, the LSK population contains LT-HSCs, ST-HSCs, multipotent progenitors (MPPs), and lymphoid primed progenitors (LMPPs) 21,30–34. By day 7 postinfection, the numbers within the LSK population that were LT-HSCs and ST-HSCs increased, whereas changes in the MPPs and LMPPs were less pronounced (Fig.5A).

The LT-HSC population contains the most immature and quiescent HSCs. CD34 and Flt3, and double-negative LT-HSCs can be subdivided further based on their expression of the signaling lymphocyte activation markers (SLAMs) CD150 and CD48 into quiescent (LSK CD150^+^CD48^−^) and more active (LSK CD150^+^ CD48^+^; CD150^−^CD48^+^) populations 35–38. There was a pronounced expansion in total LT-HSCs (Fig.5A). This increase is unlikely to be due to contamination by other cells, such as CMP that had upregulated Sca-1 in response to infection, as the cells were negative for CD16 and CD32 and were also CD34^−^ 30,32–34,36,38. Despite the increase in total numbers of LT-HSCs, the absolute number of quiescent CD48^−^ LT-HSCs remained remarkably stable even though a greater proportion of this and other subsets were proliferating after infection (Fig.5B and C). The increase in total LT-HSCs therefore was accounted for by the more activated CD48^+^ subsets 39, which increased by >100-fold postinfection. Thus, STm infection induces prolonged perturbations in HSC numbers, activation, and proliferation.

### During STm infection CLPs are found within the c-Kit^int^Sca-1^hi^ LSK^int^ population

**Figure 6:**
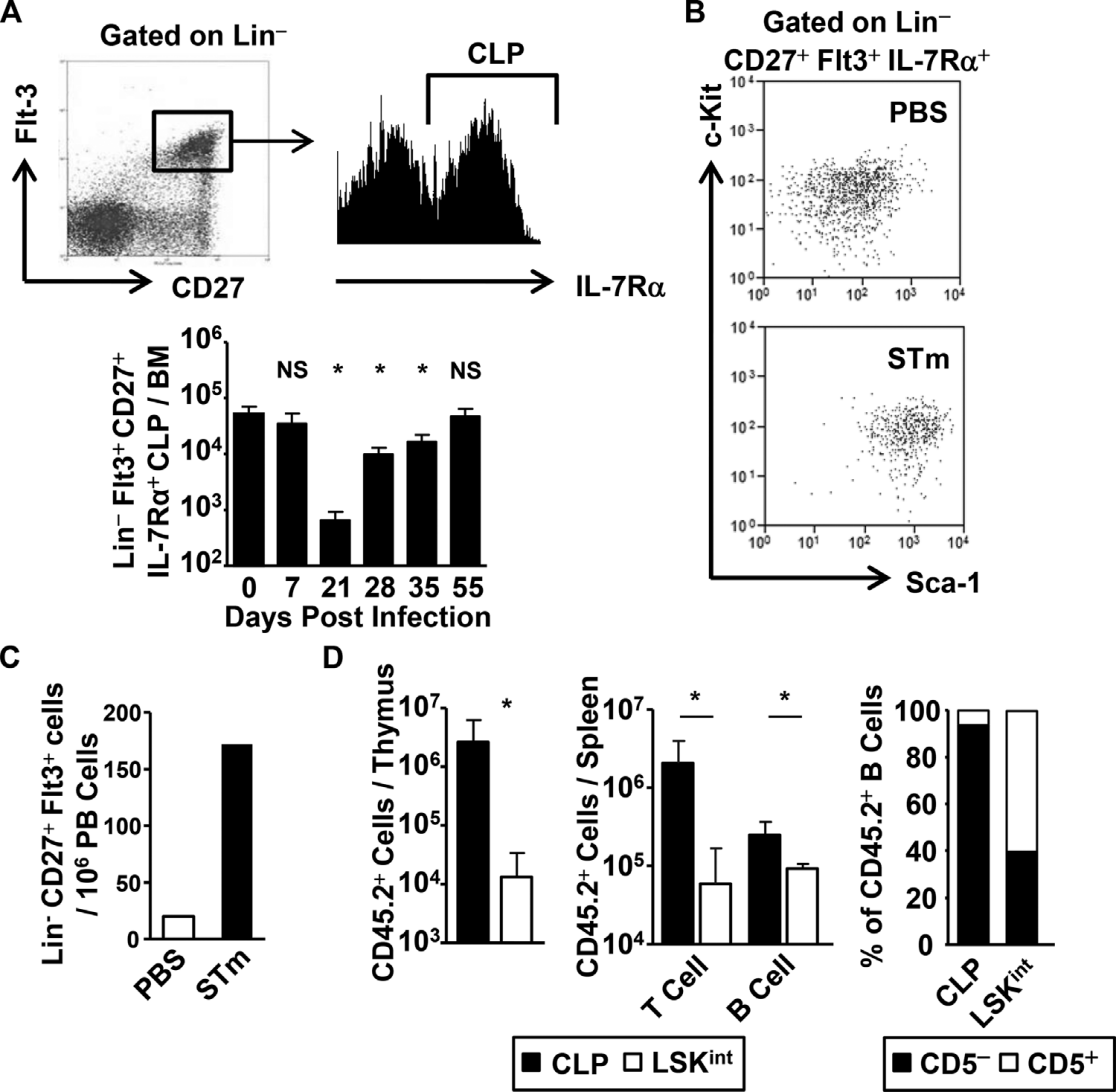
STm infection influences CLP phenotype and function. (A) Whole BM from WT mice were assessed by flow cytometry to identify Lin^−^CD27^+^Flt3^+^ progenitors, containing IL-7Rα^+^ CLPs as shown in the representative plots. Graph shows the total numbers of Lin^−^CD27^+^Flt3^+^IL-7Rα^+^ CLP progenitors in the BM during infection. (B) Representative flow cytometry plots showing the phenotypic distribution of Lin^−^CD27^+^Flt3^+^ CLPs using Sca-1 and c-Kit in BM of WT mice immunized with PBS or STm for 7 days. (C) Numbers of thymic settling progenitors in the peripheral blood of WT mice challenged with STm or PBS for 7 days were quantified by flow cytometry. Each bar represents the total number of cells found in the pooled blood from four mice and is representative of two individual experiments. (D) The reconstitution potential of 2 × 10^3^ purified CLPs or LSK^int^ cells was assessed after transfer into lethally irradiated Rag1 deficient congenic hosts. Graphs show the total number of donor progeny in the thymus, and individual subsets found in the spleen. (A–D) Data are shown as mean + S.D. (*n* = 4) and are representative of (A and B) at least three independent experiments or (C and D) two. *p ≤ 0.05 or nonsignificant (NS) compared to day 0 noninfected controls; two-tailed student's *t*-test.

LT and ST-HSCs increase during infection, yet paradoxically; numbers of classically defined CLPs fall rapidly after infection (Fig.2B). One explanation for this is that CLPs may still be present but with an altered phenotype. To examine this possibility, we used an alternative labeling strategy to detect CLPs 40, based on positive expression of Flt3, CD27, and IL-7Rα in Lin^−^ BM cells. Such a population was found in similar numbers before and at 7 days after infection, suggesting that by this method CLP numbers were similar and not reduced (Fig.6A). In contrast, numbers of CLPs identified by conventional staining were about 50-fold lower at this time (Fig.2B). Examining expression of c-Kit and Sca-1 on the Lin^−^Flt3^+^CD27^+^IL-7Rα^+^ population before and after infection showed that although c-Kit levels were similar, Sca-1 was found to be approximately 10-fold higher (Fig.6B). Therefore after infection, Lin^−^Flt3^+^CD27^+^IL-7Rα^+^ CLP cells fall within the LSK^int^ population. If CLP cell numbers were maintained after infection, then similar or elevated numbers of lymphocyte precursor cells would be expected to be detectable in the blood. Indeed, numbers of Lin^−^Flt3^+^CD27^+^IL-7Rα^+^ lymphoid progenitor cells were elevated in the blood at day 7 postinfection (Fig.6C). To confirm whether CLPs were within the LSK^int^ population during infection, we examined whether LSK^int^ cells could selectively reconstitute irradiated hosts to generate lymphocyte populations. Classical CLPs (Lin^−^IL-7Rα^+^c-Kit^int^Sca-1^int^) from naive mice or LSK^int^ cells (Lin^−^c-Kit^int^Sca-1^hi^) from day 7 infected mice were cell sorted, and 2000 of these cells were transferred into irradiated Rag1-deficient congenic hosts for 28 days (Fig.6D). In the thymus, reconstitution by LSK^int^ cells was observed but only at ∼1% of the level observed after transfer of CLPs. The reduced reconstitution of the thymus was reflected in the 30-fold lower numbers of T cells found in the spleen after transfer of LSK^int^ cells. In contrast, although splenic B-cell numbers were lower after transfer of LSK^int^ cells, this difference was small and <2-fold. Further analysis of donor-derived B-cell subsets in the spleen revealed that transferred CLPs predominantly reconstituted the B2-cell compartment (80% had a CD19^+^B220^+^CD5^−^CD21^lo^CD23^+^ follicular B-cell phenotype and 15% aCD19^+^B220^+^CD5^−^CD21^hi^CD23^−^ marginal zone B-cell phenotype, data not shown). In contrast, after transfer of LSK^int^ donor cells >50% of B cells had a B1a-like cell phenotype (CD5^+^B220^+^CD19^+^CD21^−^CD23^−^). Of the remaining nonB1a-like cells, approximately two-thirds had a follicular B-cell phenotype and the rest were marginal zone B-cells (data not shown). Therefore, although the LSK^int^ population has CLP-like potential, it is more efficient at generating CD5^+^ B lymphocytes than T lymphocytes.

### The potential and fate of LSK and LSK^−^ progenitors is altered by STm infection

**Figure 7:**
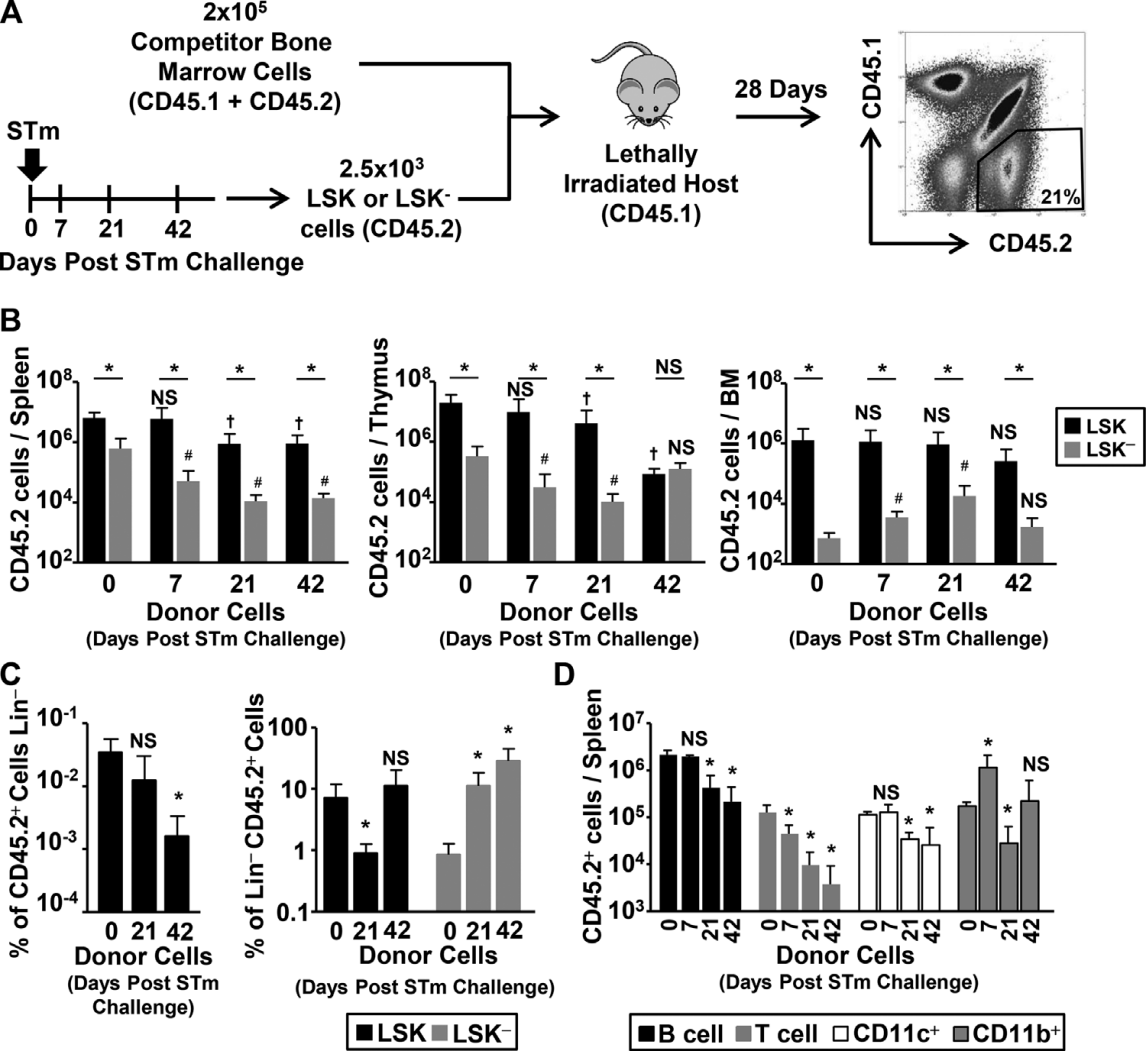
Systemic bacterial infection alters BM progenitor engraftment potential. (A) LSK or LSK^−^ cells were isolated from BM cells of WT donors exposed to infection for 7, 21, or 42 days, or noninfected (day 0) controls. Progenitors were transferred with unfractionated BM, and engraftment in irradiated congenic hosts was assessed by flow cytometry after 28 days. (B) Graphs show absolute numbers of CD45.2 progenitor progeny recovered from the spleen, thymus, and BM from LSK (black bars) or LSK^−^ (gray bars) donor cells. (C) The proportion of CD45.2^+^ BM progeny from LSK donors that reconstituted the host BM Lin^−^ compartment was assessed (left graph). The proportion of the CD45.2^+^Lin^−^ cells with an LSK or LSK^−^ phenotype was quantified (right graph). (D) Absolute numbers of mature progeny in the spleen derived from donor LSK cells exposed to infection for various periods of time in the spleen were evaluated by flow cytometry. (B–D) Data are shown as mean + S.D. (*n* = 4) and are representative of at least two independent experiments. In (B), *p ≤ 0.05 or nonsignificant (NS) between groups; ^†^*p* ≤ *0.05* or ^#^*p* ≤ 0.05 or NS compared to day 0 donor cells (two-tailed student's *t*-test). In (C and D) *p ≤ 0.05 or NS compared to day 0 donor cells (two-tailed student's *t*-test).

The capacity of infection to alter the phenotypic distribution of Lin^−^ cells led us to question whether it altered the functional activity of progenitor populations. In particular, LSK^−^ cells have been shown to be a population that accumulate with age with an incompletely understood function, with one putative role as progenitors, particularly for B cells 26–29. To do this, we examined how LSK and LSK^−^ cells isolated before and at different times after infection behaved when transferred into an irradiated noninfected host. LSK and LSK^−^ progenitor populations were sorted from noninfected (day 0) or STm infected donors on days 7, 21, and 42 postinfection. Of these, 2500 were mixed with 2 × 10^5^ whole congenic BM cells and transferred into lethally irradiated recipient WT mice (Fig.7A). On day 28 post-transfer, the progeny of transferred cells were assessed in the spleen, thymus, and BM. As previously reported 27,28, LSK^−^ cells from noninfected mice had substantially fewer progeny compared to the LSK population in the spleen (tenfold), thymus (40-fold), and BM (2000-fold) (Fig.7B). Transfer of LSK^−^ cells from infected mice showed a complex picture. Although progeny numbers in the BM were comparable or greater than day 0 LSK^−^ cells, in the thymus and spleen numbers were reduced relative to transfer of LSK cells from noninfected donors (Fig.7B).

Transfer of LSK cells from mice infected for 21 days or more resulted in lower numbers of progeny in the spleen and thymus compared to LSK cells from noninfected mice (Fig.7B). In contrast, no significant difference was observed in the BM. Although this initially appeared to suggest that LSK progenitors from infected donors persist normally in the BM, assessment of BM cells in these recipients showed that a lower proportion of BM cells from infected donors were Lin^−^, i.e. the most immature population (Fig.7C). Furthermore, although the proportion with a LSK phenotype fell in hosts receiving day 21 infected LSK donor cells, the proportion with a LSK^−^ phenotype was dramatically higher; rising from <1% of naive cells to approximately 10% from day 21 infected cells and >30% of day 42 infected cells. These data show that infection can influence the number of progeny derived from LSK cells after transfer into new hosts and the phenotype of Lin^−^ BM cells.

Finally, we assessed the fate potential of transferred LSK cells. This showed that the numbers of donor-derived lymphoid and myeloid cell numbers detected in the spleen was influenced by when after infection the LSK cells were isolated (Fig.7C). Myeloid cell numbers were less affected than lymphoid cells, which showed a clear decrease related to when after infection LSK cells were isolated, such that recovered T and B cells were 30- and tenfold lower respectively when LSK cells from day 42 infected mice were transferred (Fig.7D). Thus infection can alter the reconstitution potential of LSK progenitor cells dependent upon their length of exposure to STm.

## Discussion

In this study, we have used a systemic, resolving model of STm infection to investigate how infection can impact upon BM homeostasis. We demonstrate the phenotypic flexibility of BM progenitors after infection such as the transient increase in the numbers of LSK and LSK^−^ cells. These profound changes could persist for nearly 2 months after infection, although the phenotype of progenitor cells did eventually return to a state similar to that of resting mice. Of most potential significance is that LSK cells isolated at later time-points after infection have an altered engraftment potential and a diminished capacity to develop into lymphocytes. Such an alteration in BM progenitor phenotypes has been described during aging 19,20. This demonstrates that the BM is a highly adaptable environment that readily remodels during times of physiological stress.

Why does STm infection have such an impact on the BM? It is unlikely to be due to direct infection of BM progenitors as we were unable to culture bacteria from cell-sorted progenitor populations (data not shown). Furthermore, the most pronounced alteration in the ability of LSK cells to reconstitute effectively was seen when LSK cells were isolated from donors 42 days after infection. At this time, bacteria are no longer detected in the BM. Reflecting previous reports 4,5,7,23–25, there was a clear role for IFN-γ in modulating these processes in the BM after STm infection 41. Such a cellular and anatomical hyporesponsiveness in the absence of IFN-γ has been seen in other anatomical sites after STm; for instance, IFN-γ signaling is required for the generation of inflammatory foci in the liver 42. Despite a clear role for IFN-γ, it remains unknown whether the effects of IFN-γ are direct within the BM or indirect due to activities in peripheral sites such as the spleen or liver. Using an IFN-γ reporter mouse where eYFP is expressed under the *IFNG*-promoter, we could detect IFN-γ producing cells in the BM 24 h after infection 43 and that the cells that produced IFN-γ change over the first week of infection. One day after infection, the eYFP signal was predominantly found in the NK and NKT populations. This is compatible with their known capacity to respond rapidly to infection 44,45. By day 7 after infection, the eYFP signal was more restricted to CD4^+^ T cells and myeloid cells. Although CD4^+^ T-cell priming is rapid in this model 14,46, a substantial expansion in the numbers of T cells in other tissues is known to take a few days 15, and therefore finding multiple sources of IFN-γ during the early response is not unexpected. It is unlikely the Lin^−^ progenitor populations contribute to the earliest, local production of IFN-γ since the numbers of these cells producing IFN-γ at day 1 was similar to that in noninfected controls. Nevertheless, there was an unexpected increase in eYFP signal detected on day 7 of infection in the LSK^−^ population, and the role of this cytokine in the LSK^−^ population remains to be elucidated.

Total BM cellularity did not alter by more than twofold postinfection. Nevertheless, this disguises profound differences within the constitution of the BM compared to naive mice. The total size of the hematopoietic BM progenitor pool remained similar after STm infection, although the phenotype of cells within it was altered radically. Changes in BM progenitor populations after infection with *Ehrlichia* 7, *Plasmodium* 23, mycobacteria 5, *Escherichia coli*, 47 and microbial components 48 have been previously observed in other studies. Some of these changes are similar among these infections, whereas others differ. For instance, in models of mycobacterial and *Ehrlichia* infection followed over a period of weeks then LSK cell numbers increase and have a diminished engraftment potential. By 48 h after *E. coli* infection there is an acute expansion in LSK cells, but this was not followed for longer. Such an increase in LSK cell numbers was observed after STm. What comes across most clearly is that there are differences in the response of the LSK^−^ population among the different experimental infections. After *Ehrlichia* and *E. coli* infection there is a reduction in LSK^−^ cell numbers, whereas after STm there is an increase. Although it is not clear why different infections should have different effects, the reasons may include the level of bacteraemia induced, the level of colonization of the BM, the cellular tropism of the pathogen, the balance between intracellular and extracellular life style, and the type of protective immunity induced. This indicates that even related organisms may have a pathogen-specific signature in the BM. In this study single immunization with LPS could recapitulate some of the effects of STm infection but only in a transient manner, and this was with an amount of LPS that is far greater than that present during an infection, probably by several orders of magnitude. Nevertheless, chronic administration of approximately a third of the amount of LPS used here resulted in mild changes (<2-fold) in progenitor population numbers with the exception of quiescent LT-HSCs that were dramatically increased 48. Nevertheless, loss of TLR4, the major LPS-responding TLR, did not impair the expansion of LSK and LSK^−^ cells after STm.

Our work enhances earlier studies by examining the functional capacities of multiple progenitor populations isolated from donor mice at different times after infection. The central point is that STm infection drives an increase in Sca-1 on Lin^−^ cells, something described previously after immunization with other nonviable, proinflammatory mediators 48. One possibility is that there is a convergence between different progenitor subsets as Sca-1 expression increases. This seems unlikely to account in a large part for the changes described here. For example, there was a >200-fold loss in CMP progenitors on day 7 after infection, but only a >10-fold increase in LSK cells. This means that even an increase in Sca-1 on CMP could not account for the changes observed in this one population. Furthermore, detailed examining of progenitor cells shows that not all subsets were similarly affected by infection. During hematopoiesis, Sca-1 expression decreases as cells commit to lymphoid or myeloid progenitors 30,49. This may mean that during infection the increase in numbers of Sca-1^hi^ cells is due to an accumulation of more immature progenitors. This was clearest for CLPs, where the LSK^int^ population shared most of the phenotypic markers found on CLPs yet expressed a higher level Sca-1.

Despite the changes in the stem cell compartment and the increase in proliferation of CD150^+^CD48^−^ LT-HSCs, the total numbers of these cells remain tightly regulated. This suggests that the osteoblastic niche for these cells may not be overtly affected by infection. The stability of CD150^+^CD48^−^ LT-HSC numbers in parallel with increased proliferation may reflect faster differentiation into downstream progenitors, as seen after continuous exposure to bacterial proteins or TLR ligands 50.

LSK cells from naive and infected mice could reconstitute the BM to a similar level. Nevertheless, transfer of LSK cells from infected mice generated reduced Lin^−^ numbers, and there were differences in the numbers of LSK and LSK^−^ population's postreconstitution. This may be important as infection only altered the capacity of LSK progeny to repopulate the spleen and thymus when LSK cells were isolated from donors infected for 3 weeks or more. Also, the distribution of progeny between myeloid and lymphoid lineages in the spleen was influenced by when after infection LSK cells were isolated. This may indicate that extrinsic, environmental cues in the BM after infection influence LSK cell programming and alter their ability to repopulate compared to LSK cells from noninfected mice. Thus, restoration of normal progenitor function may require signals from both LSK cells and their environment. Alternatively, it is possible that infection can directly skew BM progenitor activity, resulting in a diminished capacity to generate lymphoid cells (Fig.7). These possibilities are not mutually exclusive. Finally, these effects are unlikely to be due to carry over of bacteria, since at day 42 only one in 10^6^ BM cells is infected with STm and cells were pretreated with antibiotics before transfer. The impact of infection on the impaired potential of LSK cells to develop into lymphoid cells is striking, yet parallel observations are made in aged BM 18–20. As we age, HSC numbers remain relatively constant, yet there is a reduction in the ability of the BM to support lymphopoiesis, with an increase in myeloid progeny development (recently reviewed in 51). The findings here may suggest that repeated episodes of infection may contribute to this phenomenon. Further assessment in aged mice that have been previously exposed to multiple, resolving infections is required to address this.

Differentiation of LSK cells into CLPs is a commitment step on the pathway to generating lymphocytes. Classically defined CLP numbers fell after infection, whereas LSK^int^ numbers increased. CLPs and LSK^int^ cells had a similar phenotype but had differing capacities to reconstitute recipient mice. Transferred donor LSK^int^ cells were poor at reconstituting T cells compared to CLPs. Nevertheless, they were better at reconstituting the B-cell compartment, particularly cells with a B1a-like phenotype. Since B1a cells have the ability to self-renew, it is possible that the numbers of B cells seen after transfer of LSK^int^ cells is through a combination of their ability to make B cells and the proliferation of these cells in the periphery. This surprising finding is now a focus of ongoing research in the group.

The expansion of LSK^−^ populations is observed after other infections 4–6,23. We observe a significant expansion of the CD25^−^LSK^−^, but not CD25^+^LSK^−^ subset. Since STm infection increases the proportion in cell cycle, it suggests that this is a direct consequence of this exposure to the pathogen. Transfer experiments showed the LSK^−^ population isolated from noninfected mice contains a heterogeneous mix of lymphoid and myeloid precursors with a lower engraftment efficiency compared to LSK cells 27,28. Transfer of infection-experienced LSK^−^ cells exposed to STm for more than 21 days reduced their reconstitution potential and prevented a full assessment of their progeny. Data, using LSK^−^ cells exposed to STm for 7 day, showed they had a heterogeneous potential, with a diminished myeloid potential and increased B-cell potential (data not shown). Therefore, the LSK^−^ population may make a transient, but limited, contribution to leukocyte numbers in the periphery during times of extreme physiological stress when their numbers are increased.

In conclusion, this study demonstrates the flexibility of the BM compartment to respond and adapt to systemic infection and that the fate potential of progenitors is influenced by the stage of infection. These findings may have implications for the generation of collections of stem cells from donors who have recently been exposed to bacterial infection and the success of their use in subsequent stem cell transplant therapies.

## Materials and methods

### Mice, bacteria, and immunization protocols

Mice used in this study were housed and used under procedure in accordance with UK Home Office guidelines under project license number 30/2850. C57BL/6 mice were obtained from Harlan. Congenic CD45.1 and CD45.1/CD45.2, and TLR4KO mice were from colonies already established at the Biomedical Services Unit, University of Birmingham. IFN-y-eYFP (GREAT) reporter mice 43 were from Jax. TLR4 deficient mice were obtained from Oriental Bioservice (Kyoto, Japan). Rag1, IFN-γ, and TNF-αR1 and two deficient mice were generously provided by Prof. Richard Grencis, University of Manchester from Jackson Laboratories. Mice were infected i.p. or i.v. where stated with 5 × 10^5^ live attenuated STm strain SL3261 taken from log phase cultures as described previously 11,52. Purified STm LPS was purchased from Alexis Bioscience. At indicated time-points, spleen, thymus and both hind legs were taken, and analyzed by flow cytometry and bacterial culture.

### Flow cytometry

Single cell suspensions were prepared as previously described 15, and contaminating erythrocytes were lysed using ammonium chloride buffer where appropriate. Cell suspensions were stained with optimal dilutions of antibodies directly conjugated to either Biotin, FITC, PE, PE-Texas Red, PE-Cy5.5, PerCP-Cy5.5, PerCP-eFluor710, PE-Cy7, Pacific Blue, eFluor 450, allophycocyanin, Alexa Fluor 647, or allophycocyanin-eFluor 780. Biotinylated antibodies were visualized using streptavidin PE-Texas Red (BD Bioscience). Lineage positive cells were gated out of whole BM suspensions using mouse hematopoietic lineage eFluor450 cocktail (eBioscience).The following specific antibodies were used to detect cell surface antigens: CD117/c-Kit (2B8), Sca-1/Ly6A/E (D7), CD127/IL-7Rα (A7R34), CD34 (RAM34), CD135/Flt3 (A2F10), CD25 (PC61.5), AA4.1 (AA4.1), CD27 (LG.7F9), CD150 (mShad150), CD48 (HM48-1), CD244.2 (eBio244F4), CD45.1 (A20), CD45.2 (104), GR-1/Ly-6G (RB6-8C5), CD11b (M1/70), CD11c (N418), CD45R/B220 (RA3-6B2), CD19 (eBio1D3), CD3 (145-2C11), CD4 (GK1.5), and CD8 (53-6.7). All antibodies were obtained from eBioscience. The CD1d PBS-57 tetramer was obtained from the NIH Tetramer Core Facility (Emory University, Atlanta, USA).

### Quantification of progenitors in peripheral blood

Progenitors in the peripheral blood were identified and quantified using a protocol adapted from Serwold et al. 40. Peripheral blood from six animals per group was collected in EDTA by terminal cardiac bleed and pooled. Blood was diluted 1:1 in RPMI and mononuclear cells enriched by centrifuging through a layer of Lympholyte-Mammal (Cedarlane Laboratories, Burlington, USA). Cells were collected, washed, and stained for Lin markers (hematopoietic lineage eFluor450 cocktail (EBioscience) and NK1.1, CD11b, and CD19 on FITC), CD27-PerCP-eFluor710, CD127-Alexa Fluor 647, Sca-1-Pe-Cy7, CD117-allophycocyanin-eFluor780, and Flt3-PE (all eBioscience). At least 5 × 10^6^ events were analyzed by flow cytometry per sample.

### Measurement of BrdU uptake after STm infection

Mice were pulsed with 1 mg BrdU (BD Bioscience) i.p. 2 h before sacrifice. BM cell suspensions were enriched by depleting lineage positive cells using mouse hematopoietic lineage biotin cocktail (eBioscience) and streptavidin magnetic beads (Miltenyi Biotech). The percentage of BM populations incorporating BrdU was quantified by flow cytometry using the BrdU Flow Kit (BD Bioscience) in combination with surface markers to identify distinct progenitor and stem cell subsets.

### Generation of radiation chimeras

Congenic hosts (CD45.1^+^) were maintained on water containing enrofloxacin (Baytril, 5mg/Kg, Bayer Animal Health) for 1 week prior and 2 weeks postirradiation. Hosts were lethally irradiated with two doses of 450 rads 2 h apart. One hour later, a mixture of 2000–2500 cell-sorted progenitor cells (CD45.2^+^) from mice exposed to STm for various time-points was mixed with 2 × 10^5^ unfractionated congenically marked host type BM (CD45.1^+^CD45.2^+^) and transferred by i.v. injection. Transferred cells were pretreated with a combination of penicillin (100 U/mL)/streptomycin (100 μg/mL)/gentamycin (25 μg/mL) and enrofloxacin for 15 min prior to cell sorting to eliminate any contaminating bacteria. Chimeras were left to reconstitute for up to 35 days.

### Statistics

Statistics were calculated using the nonparametric Mann–Whitney sum of rank test. The *p* values were calculated using Prism5 (Graphpad Software Inc.) and *p* values of ≤ 0.05 were accepted as significant.

## Supporting Information

As a service to our authors and readers, this journal provides supporting information supplied by the authors. Such materials are peer reviewed and may be re-organized for online delivery, but are not copy-edited or typeset. Technical support issues arising from supporting information (other than missing files) should be addressed to the authors.

**Figure 1**. STm infection induces the expansion specific LSK- subsets. **Figure 2**. STm infection induces an expansion in Lin-Sca-1hi progenitor populations.
