# Supplementary material for: Resolving Salmonella infection reveals dynamic and persisting changes in murine bone marrow progenitor cell phenotype and function
Source: Eur J Immunol. 2014 Jun 11;44(8):2318–30. doi: 10.1002/eji.201344350 (PMC4209805; doi:10.1002/eji.201344350)
Supplement: Supplementary file 1 — Figure 1. STm infection induces the expansion specific LSK- subsets. Figure 2. STm infection induces an expansion in Lin-Sca-1hi progenitor populations. [file eji0044-2318-sd1.pdf]

European Journal of Immunology

Supporting Information
for
DOI 10.1002/eji.201344350

Ewan A. Ross, Adriana Flores-Langarica, Saeeda Bobat, Ruth E. Coughlan, Jennifer L. Marshall, Jessica R. Hitchcock, Charlotte N. Cook, Manuela M. Carvalho-Gaspar, Andrea M. Mitchell, Mary Clarke, Paloma Garcia, Mark Cobbold, Tim J. Mitchell, Ian R. Henderson, Nick D. Jones, Graham Anderson, Christopher D. Buckley and Adam F. Cunningham

Resolving *Salmonella* infection reveals dynamic and persisting changes in murine bone marrow progenitor cell phenotype and function

© 2014 The Authors. *European Journal of Immunology* published by Wiley-VCH Verlag GmbH & Co. KGaA, Weinheim.

Ross et al. Supplementary **Figure 1**

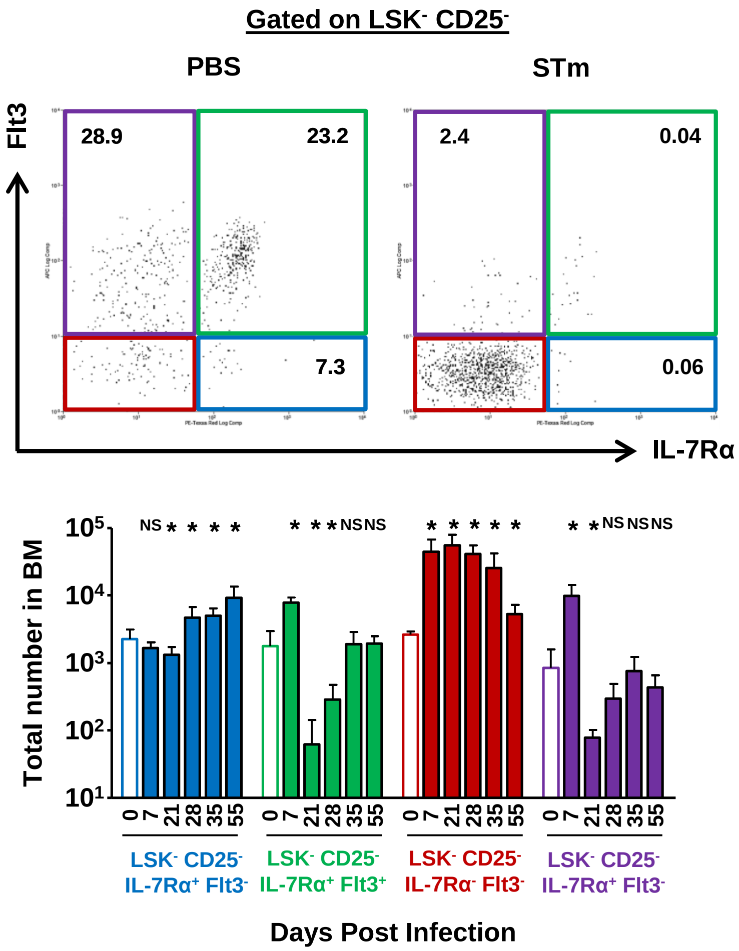

Supporting Information Figure 1. STm infection induces the expansion specific LSK$^-$ subsets.
Distinct subsets within the CD25$^-$ LSK$^-$ BM fraction were identified by expression of IL-7Rα (CD127) and Flt3 (CD135) by flow cytometry as shown in the representative plots. Changes in the absolute numbers present in the BM during infection were enumerated by flow cytometry. Data are shown as mean ± S.D. (n=4) and are representative of at least two independent time-courses. *$p \leq 0.05$ compared to day 0 non-infected control (two tailed Student's t-test).

Ross et al. Supplementary **Figure 2**

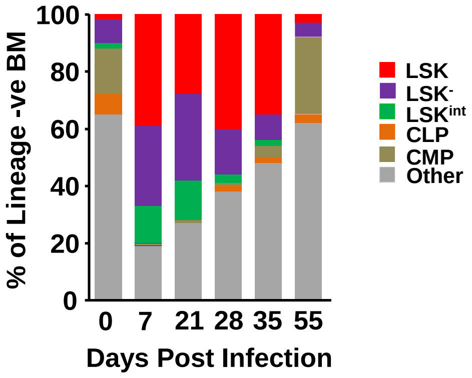

Supporting Information Figure 2. STm infection induces an expansion in Lin$^-$Sca-1^{hi} progenitor populations. The proportion of distinct progenitor subsets within the Lin$^-$ BM fraction over the time-course of infection was assessed by flow cytometry. Data are shown as mean (n=4) values of individual populations within the Lin$^-$ BM fraction and is representative of at least three independent time-courses.
